# Pregabalin versus placebo in targeting pro-nociceptive mechanisms to prevent chronic pain after whiplash injury in at-risk individuals – a feasibility study for a randomised controlled trial

**DOI:** 10.1186/s13063-018-2450-9

**Published:** 2018-01-17

**Authors:** J. Nikles, G. Keijzers, G. Mitchell, S. Schug, R. Ware, S. A. McLean, L. Connelly, S. Gibson, S. F. Farrell, M. Sterling

**Affiliations:** 1 Recover Injury Research Centre, NHMRC Centre of Research Excellence in Recovery Following Road Traffic Injuries, The University of Queensland, Herston, Australia; 2 Department of Emergency Medicine, Gold Coast Hospital and Health Service, Gold Coast, Queensland Australia; 3 School of Medicine, Bond University, Gold Coast, QLD Australia; 4 School of Medicine, Griffith University, Gold Coast, QLD Australia; 5 Faculty of Medicine, The University of Queensland, Herston, Australia; 6 School of Medicine and Pharmacology, The University of Western Australia, Perth, Australia; 7 Menzies Health Institute Queensland, Griffith University, Brisbane, Australia; 8 Institute for Trauma Recovery, Department of Anesthesiology, University of North Carolina School of Medicine, Chapel Hill, USA; 9 Centre for the Business and Economics of Health, University of Queensland, Brisbane, Australia; 10 Dipartimento di Sociologia e Diritto dell’Economia, University of Bologna, Bologna, Italy; 11 Caulfield Pain Management and Research Centre, Melbourne, Australia

**Keywords:** Whiplash-associated disorders, Pregabalin, Randomised controlled trial, Feasibility study, Motor vehicle crash

## Abstract

**Background:**

Whiplash-associated disorders (WAD) are an enormous and costly burden to Australian society. Up to 50% of people who experience a whiplash injury will never fully recover. Whiplash is resistant to treatment and no early management approach has yet been shown to prevent chronic pain. The early presence of central sensitization is associated with poor recovery. Pregabalin’s effects on central sensitization indicate the potential to prevent or modulate these processes after whiplash injury and to improve health outcomes, but this has not been investigated. This paper describes the protocol for a feasibility study for a randomised controlled trial of pregabalin plus evidence-based advice compared to placebo plus evidence-based advice for individuals with acute whiplash injury who are at risk of poor recovery.

**Methods:**

This double blind, placebo-controlled randomised feasibility study will examine the feasibility and potential effectiveness of pregabalin and evidence-based advice (intervention) compared to placebo and evidence-based advice (control) for individuals with acute whiplash injury at risk of poor recovery. Thirty participants (15 per group) aged 18–65 years with Grade II WAD, within 48 hours of injury and currently experiencing at least moderate pain (NRS: ≥ 5/10) will be recruited from Emergency Departments of public hospitals in Queensland, Australia. Pregabalin will be commenced at 75 mg bd and titrated up to 300 mg bd as tolerated for 4 weeks followed by 1 week of weaning.

**Results:**

The feasibility of trial procedures will be tested, as well as the potential effect of the intervention on the outcomes. The primary outcome of neck pain intensity at 3 months from randomisation will be compared between the treatment groups using standard analysis of variance techniques.

**Discussion:**

Feasibility and potential effectiveness data will inform an appropriately powered full trial, which if successful, will provide an effective and cost-effective intervention for a costly and treatment resistant condition. It will also have implications for the early management of other traumatic conditions beyond whiplash.

**Trial registration:**

Clinical Trials Primary Registry: Australian and New Zealand Clinical Trials Registry.

Clinical Trial Registration Number: ACTRN12617000059369.

Date of Registration: 11/01/2017.

Primary Trial Sponsor: The University of Queensland, Brisbane QLD 4072 Australia.

**Electronic supplementary material:**

The online version of this article (10.1186/s13063-018-2450-9) contains supplementary material, which is available to authorized users.

## Background

In the majority of Western countries, whiplash is the most frequent injury resulting from a motor vehicle crash (MVC) [1]. MVCs result in 50 million injuries worldwide and nearly four million emergency department (ED) consultations in the US per year [2]. In Australia, non-hospitalised minor injuries such as whiplash comprise about 72% of all survivable MVC injuries [3], with total annual costs of over $950 million [3]. The cumulative incidence of whiplash injuries resulting from MVCs has increased to over 300/100,000 people in North America and Western Europe since 1990 [4].

Up to 50% of people with whiplash injury will never fully recover [5] and up to 30% will remain moderately to severely disabled [5]. Most recovery, if it occurs, takes place in the first 2–3 months, after which time recovery plateaus [6, 7]. This indicates that early treatment will be crucial for better recovery; yet, existing early treatment approaches are inadequate. Although current clinical guidelines recommend exercise and maintaining activity for acute whiplash [8], several systematic reviews conclude that exercise/activity-based interventions provide only small effects [9–11]. Early multidisciplinary management (mainly physiotherapy and psychology) is also no more effective than usual care [12]. Behavioural interventions, such as education and advice, also demonstrate only small effects [10]. The effectiveness of medication in the early treatment of whiplash has been flagged as an urgent research need for several years [13], including at the International Summit on Whiplash Injury in 2011 [14]. Our analysis of data from the multi-year Bettering the Evaluation and Care of Health (BEACH) study of General Practitioner (GP) activity in Australia found that medications are prescribed for most whiplash cases [15], yet a recent review found no studies to support the effectiveness of any medication for whiplash-associated disorders (WAD) [16].

The most consistent predictor of poor recovery is higher initial levels of pain [17]. The early presence of pro-nociceptive mechanisms (widespread hyperalgesia, allodynia and spinal cord hyperexcitability (via nociceptive withdrawal reflexes) that does not accommodate within a few weeks) are also associated with poor recovery [18]. Some studies indicate that patients with these pro-nociceptive features do not respond as favourably to physical rehabilitation as those without these features [19]. Addressing initial pro-nociceptive mechanisms in the early acute post injury stage may improve long-term outcomes for this patient group.

Pregabalin is an obvious medication choice for this purpose. It acts to reduce central sensitization and, if shown to be effective, would alleviate concerns about prescription opioids by reducing the incidence of chronic pain, and therefore the need for long-term opioid use. The use of opioids in this patient group is a concern; indeed, the misuse of prescription opioids in the US and Canada has been described as a public health crisis, with evidence that a similar problem is developing in Australia [20]. Our research has shown that 39% of prescribed medications for WAD in general practice were opioids [15], with US data showing that early provision of opioids in the hospital ED for MVC injury patients is associated with continued use 6 weeks later [21]. Both findings highlight a potential risk for opioid misuse in this patient group.

Pregabalin, an anti-epileptic drug, reduces excitability of dorsal horn neurons after tissue damage, blocking development of pronociceptive mechanisms. It is a structural analogue of the inhibitory neurotransmitter g-aminobutyric acid, binding to the voltage-gated calcium channels, reducing the release of several excitatory neurotransmitters, and blocking the development of hyperalgesia and central sensitization [22]. Pregabalin is effective for chronic neuropathic pain conditions such as postherpetic neuralgia and painful diabetic neuropathy [23] and fibromyalgia, which has similar underlying pain processes to WAD [23]. However, treatment with pregabalin did not significantly reduce leg pain intensity or improve other outcomes in patients with mainly chronic sciatica, compared with placebo [24]. More importantly for our trial, pregabalin has been shown to prevent the development of chronic pain following acute injury, in the form of surgery. Buvanendran et al. [25] showed that administering pregabalin perioperatively and for 2 weeks postoperatively reduced the incidence of chronic neuropathic pain at 3 and 6 months after total knee arthroplasty, with less opioid consumption and better range of motion during the first 30 days of rehabilitation. Similarly, less pain and improved functional outcomes have been shown for pregabalin following spinal surgery [26, 27]. Recent systematic reviews concluded that use of pregabalin yields reductions in chronic postsurgical pain, is more effective in surgical models associated with pro-nociceptive mechanisms [28], and shows promise in preventing the transition from acute to chronic pain [29, 30]. Notably, pregabalin has positive effects on anxiety [31], indicating the potential to ameliorate symptoms of stress and arousal common after whiplash injury [32].

While surgery differs from the pain due to musculoskeletal injury in many ways, the pro-nociceptive mechanisms involved remain the same. Yet, despite this commonality, no studies have investigated effects of pregabalin following injury or trauma. Given the promising results in surgical populations, there is sufficient evidence to support the hypothesis that pregabalin used in acute whiplash injury may prevent or modulate pro-nociceptive mechanisms and improve health outcomes for this treatment-resistant condition. In light of this, we propose to test the feasibility of conducting a clinical trial of pregabalin for individuals with acute whiplash injury at risk of developing chronic pain.

Initially, we will conduct a feasibility study to hone eligibility criteria, test recruitment strategies, and develop a model for recruitment for the subsequent full scale trial, which is endorsed by the Australian and New Zealand Musculoskeletal Clinical Trials Network.

### Specific objectives

1. To evaluate feasibility by estimating expected rates of:
  i. Recruitment (number of patients approached, number consenting to participate, and number eligible to be randomised)
  ii. Missing data and participant attrition
2. To test recruitment strategies and develop a model for recruitment to a full trial
3. To identify relevant factors that could create barriers to subsequent study completion, and develop strategies to overcome these
4. To assess the potential effectiveness of pregabalin in reducing pain for patients with acute WAD at risk of poor recovery to determine the adequate sample size for a definitive full-scale effectiveness randomised controlled trial (RCT)
5. To obtain feedback from ED clinicians, local General Practitioners (GPs), trial GPs and patients on their experience with the trial and areas for improvement to inform a full-scale trial

The study will not attempt to provide evidence of clinical effectiveness for pregabalin in people with acute WAD. This is in accordance with the recommendations from the National Institute of Healthcare Research (NIHR) guidelines [33] and the Consolidated Standards of Reporting Trials (CONSORT) for the development of pilot and feasibility studies [34].

## Methods/design

### Design overview

**Fig. 1:**
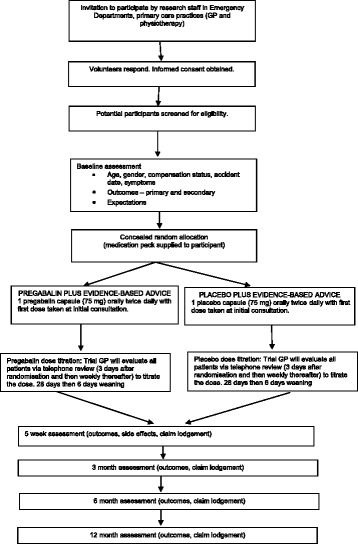
Flow chart of study design

This is a double-blind, randomised, controlled feasibility study, with 30 voluntary participants (15 per group) with acute whiplash (symptoms < 48 hours) randomly allocated to receive either pregabalin and evidence-based advice, or placebo and evidence-based advice in a 1:1 ratio, for 4 weeks followed by 6 days of weaning. Outcomes will be measured at baseline, 5 weeks, and 3, 6 and 12 months post-randomisation. Study flow is illustrated in Fig. 1.

The protocol is reported according to the Standard Protocol Items: recommendations for randomized controlled trials (Fig. 3 SPIRIT diagram and Additional file 1: SPIRIT checklist).

### Setting

Public hospitals in Queensland, Australia (Gold Coast University Hospital (GCUH) and Ipswich Hospital), and nearby General Practice clinics.

### Participants

#### Inclusion criteria

- Individuals with Grade II WAD and within 48 hours of injury
- Currently experiencing at least moderate pain (visual analogue scale: ≥ 5/10)
- Aged 18–65 years
- Proficient in written and spoken English

#### Exclusion criteria

- Known or suspected serious spinal pathology (e.g. metastatic disease of spine)
- Confirmed fracture or dislocation at time of injury (WAD IV)
- WAD III (neurological compromise, e.g. decreased reflexes, muscle power)
- Previous whiplash injury or neck pain condition requiring treatment
- Patients using gabapentin/pregabalin
- Patients with known peripheral neuropathy
- Known hypersensitivity to pregabalin use (hives, blisters, rash, dyspnea and wheezing)
- History of renal insufficiency
- Women who are pregnant or breastfeeding
- History of psychiatric illness or substance abuse
- Patient Health Questionnaire (PHQ-2) of 3 or more [35]
- Inability to speak and write in English

### Recruitment and procedure

Recruitment will be from EDs of public hospitals in Queensland, Australia. Research staff will regularly check the Emergency Department Information System (EDIS) and identify any potentially eligible patients. They will liaise with an ED doctor to ensure the patient’s history and screening results are clear for study commencement. Eligible participants who present at ED when research staff are present will complete informed consent documentation after discussion with the ED doctor, fill out baseline measures, including expectations of recovery, and then be randomly allocated to pregabalin plus evidence-based advice or placebo plus evidence-based advice.

If participants present at times when no research staff are in attendance, they will be asked by an ED doctor or nurse to provide written consent for the research team to contact them as soon as is practicable. If, when contacted, these patients agree to be involved in the trial, they will be screened at a local GP clinic, provide informed consent after discussion with the GP, and then be randomised and provided with the first medication dose.

After discharge from ED, the trial GP will evaluate all patients via telephone review (3 days after randomisation and then weekly thereafter) to titrate the dose based on the patient’s tolerance and any adverse effects, and to promote retention. Participants will be offered an optional GP visit during the active intervention phase. Participants will also be able to contact the trial GP if they have questions regarding the medication dose or any side effects.

Strategies for reaching target sample size include employing casual staff to monitor EDIS for as much of the 24 hour day as possible, using a permission to contact process for patients arriving in ED when there are no research staff, regularly reminding ED nurses and clinicians about the trial, and maintaining a high profile of the trial in the ED.

### Randomisation

Participants will be randomly allocated to either treatment group. The randomisation codes will be generated by the study statistician using computer-generated random numbers. The statistician will provide a randomisation list in variable block sizes of 4–6 for the whole trial to the study dispensing pharmacy, unavailable to those who enrol participants. To balance potential confounders and to conceal allocation, there will be separate randomisation schedules for each site. The randomisation schedule, containing patient initials, date of birth and randomisation code, will be kept in a sealed envelope in a locked filing cabinet in the pharmacy, and will be accessible after hours in case unblinding is needed.

Study medication will be prepared according to the randomisation schedule by a pharmacist not involved with data collection, then sealed in an opaque medication kit and stored in a locked drug room in the ED or at the General Practice. Allocation will occur immediately following baseline assessment. The doctor or nurse (blinded) will select the next kit in the box, record the participant’s randomisation number and provide the sealed medication kit to the participant (blinded). This will randomise them to one of two groups – pregabalin or placebo – and ensure concealed allocation and blinding of research staff, trial GPs, participants, care providers, outcome assessors and data analysts. Participants will be considered to have entered the study when the kit is opened.

### Study interventions

Using placebo as a comparator will allow us to compare pregabalin plus evidence-based advice with evidence-based advice only. All patients will receive a self-help resource to aid recovery (evidence-based advice), which describes the most effective interventions currently available (reassurance and an active physical regime including exercises) [36], ensuring no patient is without any treatment.

#### Evidence-based advice

On randomisation, an evidence-based advice booklet *Whiplash Injury Recovery: A Self Help Guide (2*^*nd*^*edition*) [36], based on recommendations of the current Australian Guidelines for Whiplash Management, will be given to all patients. It provides information about whiplash, assurance about prognosis, advice to stay active and resume working, information on correct posture and resuming functional daily activities and an exercise program proven to reduce neck pain.

#### Pregabalin or placebo

Pregabalin and placebo identical in size, appearance, smell, taste and weight (Avicel^®^) will be encapsulated by a compounding pharmacy to ensure blinding of research staff, participants, ED staff and trial GPs.

##### Dose titration

**Fig. 2:**
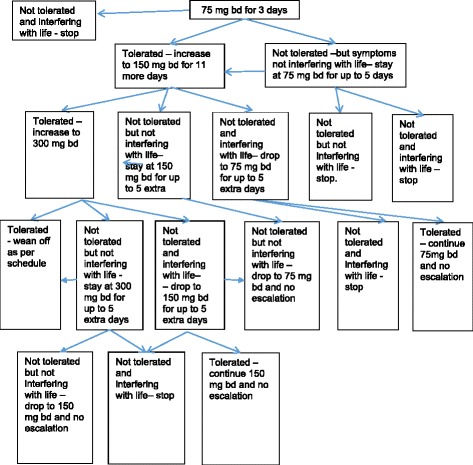
Dosing algorithm for pregabalin/placebo

Patients will receive one pregabalin capsule (75 mg) or one placebo capsule orally twice daily starting immediately on randomisation. Figure 2 delineates the dose escalation/reduction/modification regimen, which is standard clinical practice, and has also been used in previous pregabalin trials [37, 38].

##### Rationale for duration of treatment

There are no firm guidelines on treatment duration. Surgical studies have used pregabalin for 10 days to 2 weeks post-operatively [30]. For chronic neuropathic pain, 2–4 weeks of treatment is recommended [39]. In a recent trial, patients with sciatica were treated for up to 8 weeks [25]. To avoid missing benefit we will give 28 days of treatment before weaning.

### Concomitant medications

Participants will be asked not to seek other treatments and where possible not to change current medications, and GPs will be asked within reason to refrain from referring or suggesting additional or alternative treatments. All participants will maintain diaries in which they will record information about other treatments and medication taken. During the trial, GP’s calls to patients, all medications and doses will be recorded, including prescription and over-the-counter medications and natural health products, to be included in the analysis. At the end of 5 weeks, participants will be permitted to seek further treatment (e.g. additional medication, physiotherapy, etc.) if required, information about which will be recorded in cost diaries during follow-up.

### Breakthrough medication

Participants who experience high levels of continuing or worsening pain will be able to return for review by the trial GP. If there is debilitating continuing and worsening pain or continuing high levels of pain that have not improved after 7 days of treatment, despite following the trial regimen, rescue medication (Paracetamol 1000 mg qid prn or oxycodone 5 mg prn if paracetamol is not sufficient) will be provided by the trial GP. These medications are consistent with current clinical practice guidelines for WAD management [8]. Ancillary and post-trial care will be provided by the patient’s GP.

### Adherence to study medication

Adherence will be assessed by (1) daily self-recorded medication intake, (2) counts of returned tablets following treatment completion and (3) the trial GP will ask about adherence during telephone-based reviews starting at 3 days post randomisation. Adherence will be encouraged during weekly phone calls to participants. There will be full accountability for all drugs given to patients.

### Study outcome measures

- Primary outcome
  1. Average neck pain intensity over the last 24 hours at 3 months post randomisation measured using the Numeric Pain Rating Scale (NRS) [40] of 0–10.
- Secondary outcomes: feasibility
  1. Proportion of screened patients eligible
  2. Proportion of eligible patients enrolled
  3. Enrolment rate (i.e. number of enrolments per month per site)
  4. Protocol compliance
  5. Logistic model for recruitment to a full trial, including staffing requirements, and strategies to overcome any barriers identified, for example, recruiting through GP practices using the ‘permission to contact’ system for patients who have presented to ED, if recruitment is easier than through EDs directly
  6. Feedback from ED clinicians, local GPs, trial GPs and patients on their experience with the trial and areas for improvement, to inform a full scale trial
- Secondary outcomes: clinical
  1. Neck pain intensity (NRS 24 hours) at 5-week and 6- and 12-month follow-ups post randomisation
  2. Neck Disability Index (NDI) [41] at 5 weeks, 3, 6 and 12 months to measure disability
  3. Pain Catastrophising Scale (PCS) [42] at 5 weeks, 3, 6 and 12 months to measure pain catastrophising
  4. Post-traumatic Stress Diagnostic Scale (PDS) symptom score [43] at 5 weeks, 3, 6 and 12 months to measure post-traumatic stress symptoms
  5. Depression, Anxiety and Stress Scale (DASS) [44] at 5 weeks, 3, 6 and 12 months
  6. Generic measure of health status scores (SF-12) [45] at 5 weeks, 3, 6 and 12 months
  7. Proportion of patients who lodge a compensation claim at 5 weeks, 3, 6 and 12 months
  8. Number of doses of breakthrough medication taken measured at 5 weeks, 3, 6 and 12 months
  9. Number of adverse events compared between treatment groups
  10. S-LANSS [46] during follow-up from baseline, compared between treatment groups

Outcome measures have established reliability and validity, are recommended by the International Whiplash Summit [14] and the Bone and Joint Decade Neck Pain Task Force [47], and follow IMMPACT recommendations for design of clinical trials for chronic pain prevention [48].

**Fig. 3:**
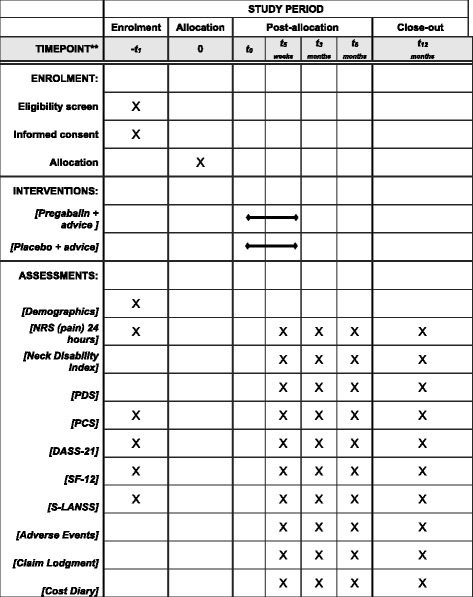
SPIRIT diagram

The Recommendations for Interventional Trials (SPIRIT) figure demonstrates the timing of the interventions and outcomes (Fig. 3).

### Sample size

Since this is a feasibility study, a sample size calculation was not performed. We will aim for 30 participants (15 in each arm) as this will be a large enough sample to inform about the practicalities of delivering the intervention in the ED, recruitment, uptake and attrition. The results of this study will inform any necessary post-trial modifications or remodelling prior to implementation of a larger scale multisite RCT.

### Loss to follow-up

This feasibility study aims to assess attrition rates for a future large RCT. In order to reduce attrition, we will aim to foster trusting relationships via weekly telephone calls to help participants feel engaged in the study. This will also improve adherence to intervention protocols. If participants discontinue or deviate from intervention protocols, we will attempt to collect at least the primary outcome.

### Unblinding

The Principal Investigator will be able to unblind individual cases if any of the following criteria are met:

1. Emergency unblinding – to make a clinical treatment decision or when an unexpected serious adverse event occurs
2. At the request of the Data Safety Monitoring Board
3. At the conclusion of the study to determine the potential effect of intervention

If a medically qualified investigator requires identification of the trial medication composition, they must quote the randomisation code, participant initials and date of birth to the Principal Investigator, who will contact the pharmacy and quote the randomisation code and trial title. The pharmacy will unblind for the specific participant only. The medically qualified investigator will talk to and unblind the participant.

### Data management

This trial will be conducted in accordance with ICH Guidelines for Good Clinical Research Practice [49] and relevant local ethical regulations. Study data will be collected and managed using a regulatory approved electronic data capture system (REDCap) hosted at UQ [50], on the UQ server. REDCap is a secure, web-based application designed to support data capture for research studies, that will house all clinical and safety data. It will provide (1) an intuitive interface for validated data entry; (2) audit trails for tracking data manipulation and export procedures; (3) automated export procedures for seamless data downloads to common statistical packages; and (4) procedures for importing data from external sources.

Data from follow-up questionnaires will be entered by patients via email links to the REDCap database, and the remaining data by the research team. Data quality will be assured through range checks for data values. Integrity of trial data will be monitored by regularly scrutinising data for omissions and errors. In order to protect confidentiality before, during and after the trial, personal information about potential and enrolled participants will remain secure in a locked research office at GCUH or Ipswich Hospital. Study data will be retained, securely password protected, for a minimum of 15 years from completion. Details of data management procedures can be found in the protocol.

### Data analyses

Because of participant numbers and the aims of the study, we will report descriptive analyses. Recruitment rate (number of patients approached, number consenting to participate and number eligible to be randomised) will be reported, as will frequencies and proportions of missing data and participant attrition, both during intervention and follow-up periods.

A model for recruitment to the main trial will be described, including the success of various recruitment strategies, for example, is recruitment easier though EDs or through GP practices using the ‘permission to contact’ system? It will also include how the ED and general practice sites worked together, how to maximise research staffing efficiency in terms of rostering, etc. Relevant factors that could create barriers to subsequent study completion will be described, and strategies to overcome these discussed.

#### Statistical analysis

Trends in the data will be analysed by our biostatistician blinded to group allocation using intention-to-treat. We will analyse the effect of treatment separately for each outcome using mixed-effects models with random intercepts for individuals to account for correlation of repeated measures within participants. Treatment group will be included as a fixed effect. We will obtain estimates of the effect of the intervention and 95% confidence intervals by constructing linear contrasts to compare the adjusted mean change (continuous variables) or difference in proportions (dichotomous variables) in outcome from baseline to each time point between the pregabalin plus evidence-based advice and placebo plus evidence-based advice groups. Missing data will be examined for patterns of missingness.

#### Secondary outcomes

Because of participant numbers, we will present descriptive analyses of the secondary outcomes.

### Cost related data and analyses

Cost diaries, a reliable and valid tool for determining costs in cost-effectiveness research [51], will be collected at each follow-up assessment. In this study we will test the feasibility of using these diaries.

Direct costs (e.g. general practitioner, physiotherapy, chiropractor, pharmaceutical services) will be calculated using market prices estimated from Medicare Benefits Schedule, worker’s compensation scheme payment schedules and Pharmaceutical Benefits Scheme. Direct healthcare costs (e.g. consumer co-payments) and non-healthcare costs not captured by insurer payments, will be identified. Examples include other professional care, transportation costs and time spent by family members or volunteers providing care.

Indirect costs include lost economic productivity due to the injury. A shadow wage rate will be used to identify opportunity costs of time spent away from work, calculated using income and employment data collected at baseline. Utility weights will be generated using participants’ SF-12 responses, translated to SF-6D utilities [52] using a new Australian algorithm [53]. The resulting cost-effectiveness measure will be cost per QALY saved. *N*-way sensitivity analyses will model second-order uncertainty (e.g. magnitudes of probabilities, treatment effects) where appropriate.

### Monitoring

#### Collecting, assessing, reporting and managing adverse events

The most common side effects of pregabalin are dizziness and sedation; the dose will be titrated as tolerated. Leg oedema can also develop and may require discontinuation. More severe side-effects are rare.

Information about solicited and spontaneously reported adverse events will be sought from all participants during telephone reviews by the trial GP. If a participant reports an adverse event, the trial GP will determine appropriate action, which may include dose alteration or withdrawal. If a serious adverse event (SAE) is identified, the trial GP will forward this information immediately to the Principal Investigator and Data Safety Monitoring Board. All SAEs, suspected adverse reactions and serious unexpected suspected adverse reactions will be recorded immediately in the source documents, and on the adverse event case report form. Each event will be followed until resolution, stabilisation or until it has been determined that the study treatment is not causal. SAEs still ongoing at the end of the study will be followed up to determine final outcome. Any SAE occurring after the study considered to be possibly related to study treatment will be recorded and reported immediately. Compensation to those who suffer harm from trial participation will be provided by the trial sponsor.

### Withdrawals

Participants will be withdrawn if they develop any exclusion criteria or if they choose to do so, without prejudice to current or future management. The follow-up schedule will continue unchanged for all randomised participants unless a participant chooses to withdraw from follow-ups. If participants cannot complete follow-up outcome measures online or on paper, or are lost-to-follow-up and/or are non-compliant with dosing, we will employ phone calls, home visits and/or communication with their GP. Patients who withdraw during dosing will be made aware of the need for weaning from study medication.

### Dissemination

Authorship eligibility guidelines will follow ICMJE guidelines. The final trial dataset will be available to the investigative team and on reasonable request.

## Discussion

Chronic pain and disability following whiplash injuries is an enormous health and economic burden for Australia and other industrialised countries. Current treatments offer only modest benefit. This feasibility study will assess the feasibility and potential effectiveness of an innovative early intervention delivered to ‘high-risk’ individuals in EDs. Feasibility and potential effectiveness data will inform a full trial, which if successful, will provide an effective and cost-effective intervention with immediate clinical applicability for a costly and treatment resistant condition. It will also have implications for early management of other traumatic conditions.

The protocol was developed for an ‘at risk’ population by a team including a physiotherapist, pain specialist, ED clinician researcher, GP, clinical psychologist and biostatistician.

The primary effectiveness measure is neck pain intensity at 3 months from randomisation for this feasibility study. Measuring this at 3, 6 and 12 months will allow assessment of any sustained effect on outcomes. In the feasibility trial described here, the study will not be powered to assess these potential effects, but will allow necessary post-trial modifications that will maximise effectiveness of the trial to be implemented prior to the rollout of a larger scale multisite RCT.

### Strengths

This study has been designed and is being conducted in accordance with the principles of the Declaration of Helsinki (1996), Good Clinical Practice and all applicable regulatory requirements. This will increase validity, participant safety and scientific integrity as well as reduce bias. The Trial Steering Committee, which includes key study investigators, will ensure that study quality is maintained throughout.

### Limitations

Recruiting through EDs means that our cohort of patients will not be entirely representative of the entire population of whiplash patients. However, by recruiting participants through EDs, we will be most likely to enrol participants with greater pain and distress and therefore at higher risk of poor recovery – the very group we aim to target. Data from our current trials [54] would support this, where patients with acute injury recruited from ED reported average higher pain levels (6.2 ± 1.5) compared to those recruited from primary care and advertisement (4.5 ± 1.0). In addition, we aim to reduce central sensitization beginning in the early aftermath of injury, and ED recruitment allows us to recruit patients within hours of MVC.

### Trial status

Protocol version 8 is dated July 2017. Recruitment began on 20 January 2017 and is ongoing. Important protocol modifications (e.g. changes to eligibility criteria, outcomes, analyses) will be communicated to relevant parties (e.g. investigators, Human Research Ethics Committees, trial participants, trial registries, journals, regulators) as needed.

## Additional file

Additional file 1: SPIRIT checklist. (DOC 120 kb)

## Abbreviations

AE: adverse event
DASS: Depression, Anxiety and Stress Scale
ED: emergency department
EDIS: emergency department information systems
GCUH: Gold Coast University Hospital
GP: general practice
MVC: motor vehicle crash
NDI: Neck Disability Index
PCS: Pain Catastrophising Scale
PDS: Post-traumatic Stress Diagnostic Scale
SAE: serious adverse event
S-LANSS: Self-report – Leeds Assessment of Neuropathic Symptoms and Signs Pain Scale
WAD: whiplash-associated disorders
